# Fatigue in out-patients with inflammatory bowel disease: Prevalence and predictive factors

**DOI:** 10.1371/journal.pone.0181435

**Published:** 2017-07-27

**Authors:** Albert Villoria, Víctor García, Angelina Dosal, Laura Moreno, Antònia Montserrat, Ariadna Figuerola, Diana Horta, Xavier Calvet, María José Ramírez-Lázaro

**Affiliations:** 1 Servei Aparell Digestiu, Hospital Universitari Parc Taulí, Institut d’Investigació i Innovació Parc Taulí, Sabadell, Spain; 2 Departament de Medicina, Universitat Autònoma de Barcelona, Bellaterra, Spain; 3 CIBERehd, Instituto de Salud Carlos III, Madrid, Spain; 4 Grup de Recerca Consolidat (SGR01500), Barcelona, Spain; University Hospital Llandough, UNITED KINGDOM

## Abstract

**Background and Aim:**

Fatigue is a common and bothersome symptom in inflammatory bowel disease (IBD) patients. The study was aimed to determine the relationship of biological and psychological factors with IBD-related fatigue.

**Methods:**

Consecutive clinically inactive IBD outpatients receiving immunosuppressants or biological drugs were enrolled between January and December 2013. Patients completed a Fatigue score (FACIT-F), various psychological, quality of life (IBDQ-9), and IBD activity scores. Biological parameters were assessed, including levels of interleukins (IL-5, IL-8 and IL-12) and micronutrients.

**Results:**

We prospectively recruited 202 patients (28% ulcerative colitis and 72% Crohn’s disease) for the study. Fatigue measured by FACIT-F score was prevalent in the studied population (54%, 96/177) and higher than in the general population. In the univariate analysis no relation was found between IL levels or micronutrient deficiencies and fatigue. Fatigue was significantly related to female sex, Crohn’s disease, joint disorders, body mass index (BMI), psychological tests, thiopurine use, and anti-TNF treatment. All these variables were included in the multivariate analysis. Female sex (OR: 4.8), high BMI (OR:1.2) and higher depression rates (OR:1.2) were predictors of increased fatigue. High IBDQ-9 score (OR: 0.82) was significantly related to lower degrees of fatigue.

**Conclusion:**

Fatigue was prevalent in quiescent IBD patients with moderate-to-severe disease. It was associated with high levels of depression, low quality of life, and female sex. No association was found with the other biological and psychological factors evaluated.

## 1. Introduction

Fatigue is a common challenging symptom in many chronic diseases, either physical or psychiatric, such as heart failure, Parkinson’s disease, depression. Recently, a high prevalence of fatigue has been reported in chronic inflammatory diseases such as multiple sclerosis [1], rheumatoid arthritis [2], and inflammatory bowel disease (IBD) [3–5]. Some patients with these conditions continue to have severe fatigue even when inflammation is fully controlled with treatment.

The most frequent symptoms of IBD patients are diarrhea and abdominal pain. However, fatigue can be just as problematic for these patients; it may be severe and is not always explained by disease activity. In fact, IBD-related fatigue is also frequent in patients in disease remission [6].

The reported prevalence of fatigue in IBD ranges from 29% to 41% during clinical remission and from 57% to 72% during active disease [3–5]. Clinical features such as disease activity, time since IBD diagnosis [7], treatments (methotrexate, thiopurine, 5-ASA or biological) [4,7–9], and analytical parameters (hemoglobin and C-reactive protein levels) [10] have been related to fatigue. Few studies, however, have sought to as certain factors related to fatigue in IBD patients in remission.

In a previous work [11], it was hypothesized that subclinical inflammation could be the cause of fatigue. In this study, pro-inflammatory interleukins (IL) and leukocyte subsets were evaluated. Increases in IL-5, 12, memory T-cells, and neutrophils were related to increased fatigue; conversely, lower rates of fatigue were found in patients with high IL-8 [11].

In addition, multiple micronutrient deficiencies, including minerals and vitamins, have been reported in IBD [12]. Some of these deficiencies (e.g., folic acid, vitamin D, iron, niacin, or selenium) are commonly associated with fatigue [13–16]. To date, however, nutritional status has not been evaluated as a possible cause of fatigue in IBD.

The aim of this study was to investigate biological factors associated with fatigue in IBD, including the evaluation of pro-inflammatory cytokines (interleukins 5, 8 and 12) and micronutrients.

## 2. Materials and methods

### 2.1 Patients

Consecutive IBD outpatients attending the Hospital de Sabadell Gastroenterology Day Hospital for scheduled monitoring of immunosuppressant treatment (such as thiopurine or methotrexate) or infliximab infusion between January and December 2013 were eligible.

Exclusion criteria were:

1. Age below 18 years.
2. Inability to understand or complete the questionnaires
3. Refusal to give written informed consent prior to participation
4. Active infection at the time of inclusion.
5. Concomitant diseases not related to IBD (e.g., cancer, heart disease, pulmonary disease) that might contribute to the presence or the severity of fatigue.
6. Active flare of IBD in the last 3 months.

### 2.2 Methods

After receiving detailed information about the study, patients were asked to complete a set of questionnaires, and a blood sample was obtained for analysis. We recorded demographic data, IBD classification according to the Montreal classification, and history of drug administration. In addition, clinical activity of the disease was measured using the Harvey-Bradshaw score [17] for Crohn’s disease (CD) and the modified Mayo score for ulcerative colitis (UC) [18].

### 2.3 Questionnaires

#### 2.3.1. Functional Assessment of Chronic Illness Therapy-Fatigue (FACIT-F)

Fatigue was analyzed using the FACIT-F questionnaire. This questionnaire, previously validated in Spanish and for IBD patients [19], comprises 40 items divided into five subscales: Physical wellbeing (PWB), Social/family wellbeing (SWB), Emotional wellbeing (EWB), Functional Wellbeing (FWB), and Fatigue subscale. The first four subscales (27 items) evaluate general features of chronic illness, and the 13 items of the Fatigue subscale specifically focus on fatigue. Each item has five possible answers, from 0 (very fatigued) to 4 (not at all fatigued). The score of each subscale is the sum of the coded values of its items.

The Fatigue subscale (FS) was the main item used: higher scores on FS indicate less fatigue.

#### 2.3.2. Beck Depression Inventory (BDI)

The Spanish validated 21-item version of the BDI [20] was used to analyze depression. This self-administered questionnaire evaluates the clinical features of melancholy and intrusive thoughts present in depression. Each item has a different number of options (from 4 to 8), which then have to be converted into four possible values (0, 1, 2, or 3). Higher BDI scores indicate a higher grade of depression.

#### 2.3.3. State-Trait Anxiety Inventory (STAI)

To analyze anxiety, the STAI questionnaire was used [21]. This self-administered questionnaire was designed to evaluate two independent concepts of anxiety: anxiety as a state or as a trait. The evaluation of each of the concepts comprises 20 items. The STAI is useful both in the healthy population and in patients. Higher scores on the STAI indicate a higher grade of anxiety. Only the 20 state items were recorded, since active anxiety symptoms were considered more relevant for the study.

#### 2.3.4. Epworth Sleepiness Scale (ESS)

To assess sleep disturbances, the validated Spanish version was used [22]. This self-administered questionnaire measures the general level of daytime sleepiness, or subjects’ average sleep propensity in daily life. It comprises questions assessing the likelihood (from 0 to 3) of dozing off or falling asleep in different situations. Higher scores indicate a greater level of sleepiness.

#### 2.3.5. Quality of life in inflammatory bowel disease (IBDQ-9)

To study quality of life (QoL), the shortened Spanish version of the IBDQ was administered [23]. The IBDQ-9 consists of nine items assessing different aspects of life in IBD patients. Each item has seven different possible answers, scoring from 1 (poorest) to 7 (best). The straight summation of all nine items provides a direct score, which is then transformed into a final score. Higher scores correspond to a better quality of life.

### 2.4 Blood samples

Routine biochemical (creatinine, potassium, sodium, albumin, C-reactive protein (CRP), erythrocyte sedimentation rate (ESR), vitamin B12 and serum folate test) and hematological tests (complete blood count, iron, transferrin saturation index (TSI), ferritin) were performed. To evaluate micronutrients, magnesium, cooper, phosphate, zinc, calcium and 25-OH vitamin D were also determined.

Finally, interleukins IL-5, IL-12 and IL-8 were determined obtaining 5 ml of peripheral blood by venipuncture into EDTA-K3 tubes. Plasma was separated by centrifugation and frozen at -20°C until use. Samples were prepared for analysis in a 96-well plate utilizing a custom 3-cytokine Milliplex MAP Human Cytokine/Chemokine Magnetic Bead Panel (Millipore Corp., Billerica, MA) following the kit-specific protocols provided by Millipore. Interleukins were simultaneously quantified using the analytical test instrument Luminex-200, which utilizes xMAP technology (Luminex Corp., Austin, TX), and xPONENT3.1 software (Luminex). The intra-assay coefficient of variation was 7% and the interassay was 11%. The values were presented in picograms per milliliter (pg/mL).

### 2.5 Ethics

The Ethics committee of University Hospital of Sabadell approved the study. Patients gave written informed consent before participating.

### 2.6 Statistical methods

The sample size required to provide a desired precision of 5% with a confidence level of 90%, calculated considering an estimated prevalence of fatigue of 30% and 15% losses, was 217.

A descriptive analysis was performed, expressing quantitative variables as means ± standard deviation (SD) or as medians and range, and qualitative variables as frequency and percentage. Normal distribution was assessed by the application of a Kolmogorov-Smirnov test. In the comparative study, Student’s t-test for unpaired data was used for quantitative variables and the chi-square test for qualitative variables. Non-parametric tests were used when variables were not normally distributed. Univariate regression analyses were performed to identify the psychological and biological factors associated with fatigue in our group of IBD patients. After transforming fatigue from a quantitative to a qualitative variable (having/not having fatigue), a logistic regression analyses was performed and odds ratios were calculated. Variables with *p* ≤ 0.05 in the univariate analysis were included in the regression model. Statistical analysis was performed using SPSS 21 (Hewlett-Packard, Chicago, IL).

## 3. Results

### 3.1 Patients’ characteristics

Two hundred and two patients were prospectively recruited for the study. Twenty-five patients were excluded because they did not complete FACIT questionnaires, so finally 177 patients were included for the analysis. The demographic and disease characteristics of the participants are presented in Table 1. Most patients were in clinical remission or had very mild symptoms: Harvey-Bradshaw score was ≤ 5 in 90% and ≤ 7 in 98% of CD patients, and the modified Mayo score was ≤ 2 in 78% and ≤ 4 in 93% of UC patients.

**Table 1:** Demographic data of the patients.

Age (mean±SD), years	39±12
Gender (M/F)	102/75
Years of disease (mean±SD)	9±6
Body Mass Index (mean±SD)	25±4.4
Smoker (yes/no)	62/115
**Type of IBD.**	
- Crohn:	
- Ileo-colic (n,%)	(49/127, 38%)
- Colic (n,%)	(19/127, 15%)
- Ileum (n,%)	(59/127, 47%)
- Inflammatory (n,%)	(80/127, 63%)
- Stenosing (n,%)	(28/127, 22%)
- Fistulizing (n,%)	(18/127, 15%)
- Ulcerative Colitis:	
- Proctitis (n,%)	(6/50, 12%)
- Left Colitis (n,%)	(16/50, 32%)
- Pancolitis (n,%)	(28/50, 56%)
Abdominal Surgery (yes/no)	43/134
5-ASA (n, %)	(29/177, 16%)
Thiopurines (n,%)	(137/177, 77.5%)
Methotrexate (n,%)	(12/177, 6.7%)
Infliximab (n,%)	(45/177, 25.4%)
Adalimumab (n,%)	(27/177, 15.3%)

### 3.2 Fatigue prevalence and severity

The median fatigue score in the whole group was 38 points (range 1–52). Considering a score of 40 points or more as normal in the healthy population [24], the prevalence of fatigue in our group was 54% (96/177). Eleven percent of patients (20/177) had severe fatigue (FS scores < 20 points) and 43% (76/177) had mild fatigue (20 ≤ FS ≤ 40 points).

### 3.3 Factors related to fatigue

#### 3.3.1. Demographical and clinical parameters

In the univariate analysis, female patients (n = 75) were more fatigued than males (n = 105) (31±13 vs 40±11 points, mean± SD, p< 0.001). In terms of type of disease, fatigue was more prevalent in CD patients (n = 75/127, 59%) than in UC patients (n = 21/50, 42%) but the severity according to the FACIT-FS did not differ (mean FS: 25±9.2 vs 26±6.8; p<0.7) between CD and UC patients. This increased prevalence of fatigue in CD was not associated with worse clinical activity scores, longer time since diagnosis, or the presence of a fistula.

Extra-intestinal manifestations of the disease were also evaluated. Joint disorders were the only ones significantly associated with fatigue, with very low FS scores (Table 2). Treatment for IBD was also evaluated: fatigue was more prevalent inpatients not receiving thiopurines and in those receiving anti-TNF treatment (Table 2).

**Table 2:** Relationship between fatigue and biological variables: Univariate analysis.

	Fatigue Score	Significance (p)
**Gender** (M/F)	(39±11)/(30±13)	**p< 0.001**
**Smoking** (Yes/No)	(37±12)/(33±13)	p = 0.08
**Crohn/Ulcerative colitis**	(35±12)/(39±11)	**p = 0.037**
**Crohn’sPattern**		
Inflammatory	(34±13)	
Stenosing	(33±11)	
Fistilizing	(39±19)	p = 0.289
**Ulcerative Colitis**		
Proctitis	(42±5)	
Left colitis	(42±12)	
Pancolitis	(36±12)	p = 0.33
**Jointpain** (Yes/No)	(27±12)/(38±11)	**P< 0.001**
**Abdominal surgey** (yes/no)	(34±12)/(36±13)	p = 0.68
**Thiopurine** (yes/no)	(37±12)/(31±13)	**p = 0.007**
**Methotrexate**(yes/no)	(31±15)/(36±12)	p = 0.26
**TNF-inhibitors**(yes/no)	(32±12)/(38±12)	**p = 0.003**

Data are expressed in mean ± standard deviation.

Finally, BMI was significantly correlated with fatigue (r: -0.18; p = 0.01); patients with higher BMI showed lower FS scores.

#### 3.3.2. Interleukins

**Fig 1:**
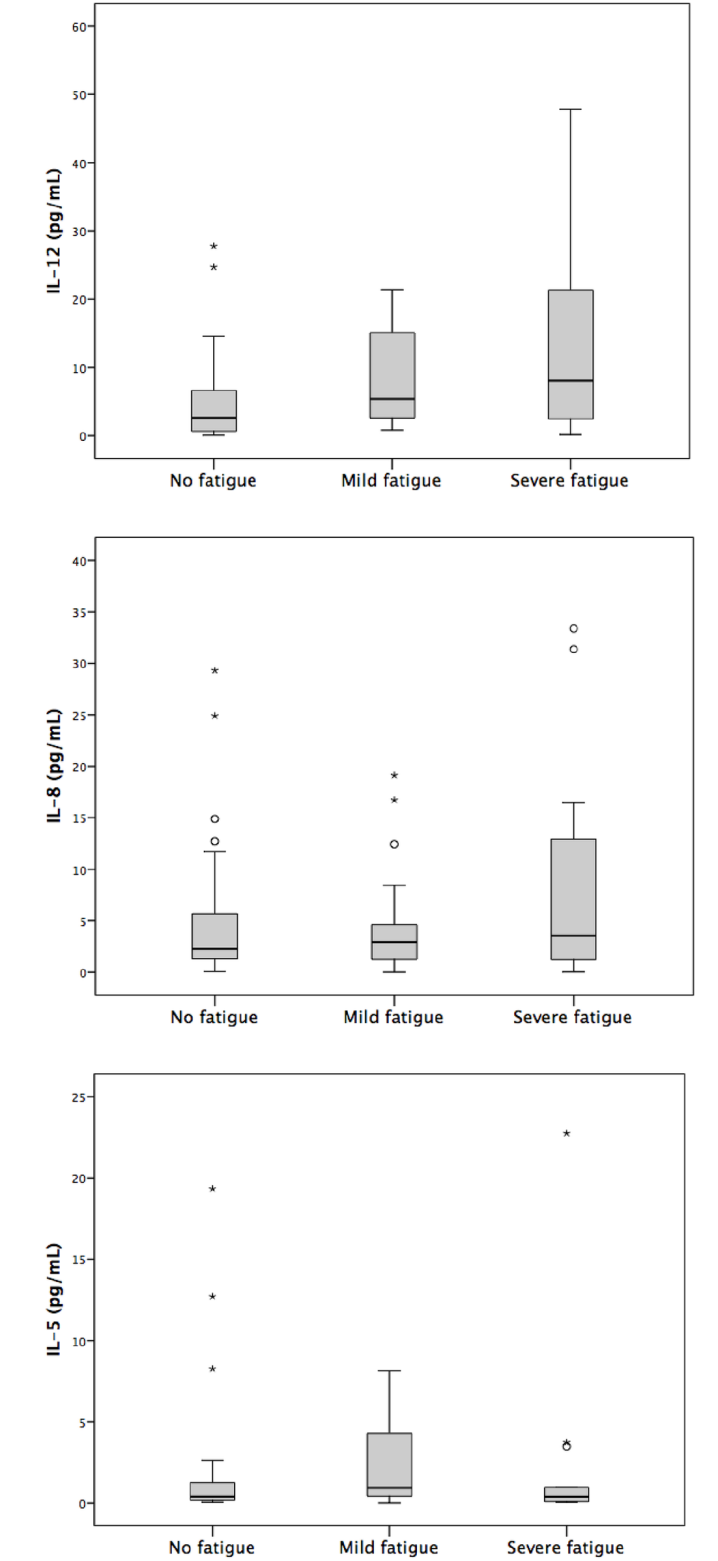
IL levels (pg/mL) in fatigue and non fatigue patients.

Despite the good sensitivity of the technique, a percentage of patients had no detectable interleukin levels (63% in IL-12, 55% in IL-8 and 34% in IL-5). In those with detectable IL, no statistical differences were seen between fatigued and non-fatigued patients and mean plasma concentration of interleukins were similar in both groups: IL-12 (7.1±13 vs 11.5±16 pg/ml), IL-8 (4.4±5.5 vs 12.1±48 pg/ml) and IL-5 (1.5±3.6 vs 7.4±32 pg/ml) (Non-fatigued Vs fatigued patients, p value > 0.05 for all the comparisons). No significant increases in IL values were observed in a subanalysis comparing severely and mildly fatigued patients with non-fatigued ones separately (Fig 1).

#### 3.3.3. Micronutrients

Mean values of all the micronutrients evaluated were within normal values (NV) except for 25 OH-vitamin D (17.5±7.3 ng/mL, NV: > 30 ng/mL) that showed a moderate deficit in the whole group. No differences in micronutrients (including vitamin D) were observed between fatigued and non-fatigued patients (see Table 3).

**Table 3:** Relationship between fatigue and biological and psychological variables: Univariate analysis.

	No Fatigue (FS>40)	Mild Fatigue (FS 20–40)	Severe Fatigue (FS<20)	Significance (p)
Age (years)	38±12	40±12	37±8	0.29
Years of disease	9±6	9±7	8±6	0.9
**Psychological test**				
Beck	5±4	14±6	18±8	**0.001**
Stai	14±7	27±10	38±11	**0.001**
Epworth	4±3	6±3	9±3	**0.001**
IBDQ-9	72±7	58±5	52±5	**0.001**
**Blood test**				
Hb(g/L)	13.8±1.3	13.6±1.3	13.3±1.6	0.16
Neutrophils(x10^9^/L)	3.8±1.6	5.3±8	4.3±2	0.18
Platelets(x10^9^/L)	254±83	261±81	269±81	0.79
CRP(mg/dL)	0.3±0.6	0.3±0.7	0.4±0.7	0.74
Ferritin (ng/mL)	131±113	134±137	85±78	0.16
**Micronutrients**				
Calcium (mg/dL)	8.7±0.5	8±0.5	8±0.5	0.86
Cooper (mcg/dL)	86±24	87±24	101±30	**0.04**
Phosphate (mg/dL)	3.2±0.5	3.1±0.5	3±0.4	0.18
Magnesium (mg/dL)	1.8±0.2	1.8±0.1	1.7±0.2	0.63
25-OH vit D (ng/mL)	17.4±7	17±8	18±7	0.90
Zinc (mcg/mL)	78.6±12	76±12	75±12	0.38

Data are expressed in mean ± standard deviation.

#### 3.3.4. Psychological tests

**Fig 2:**
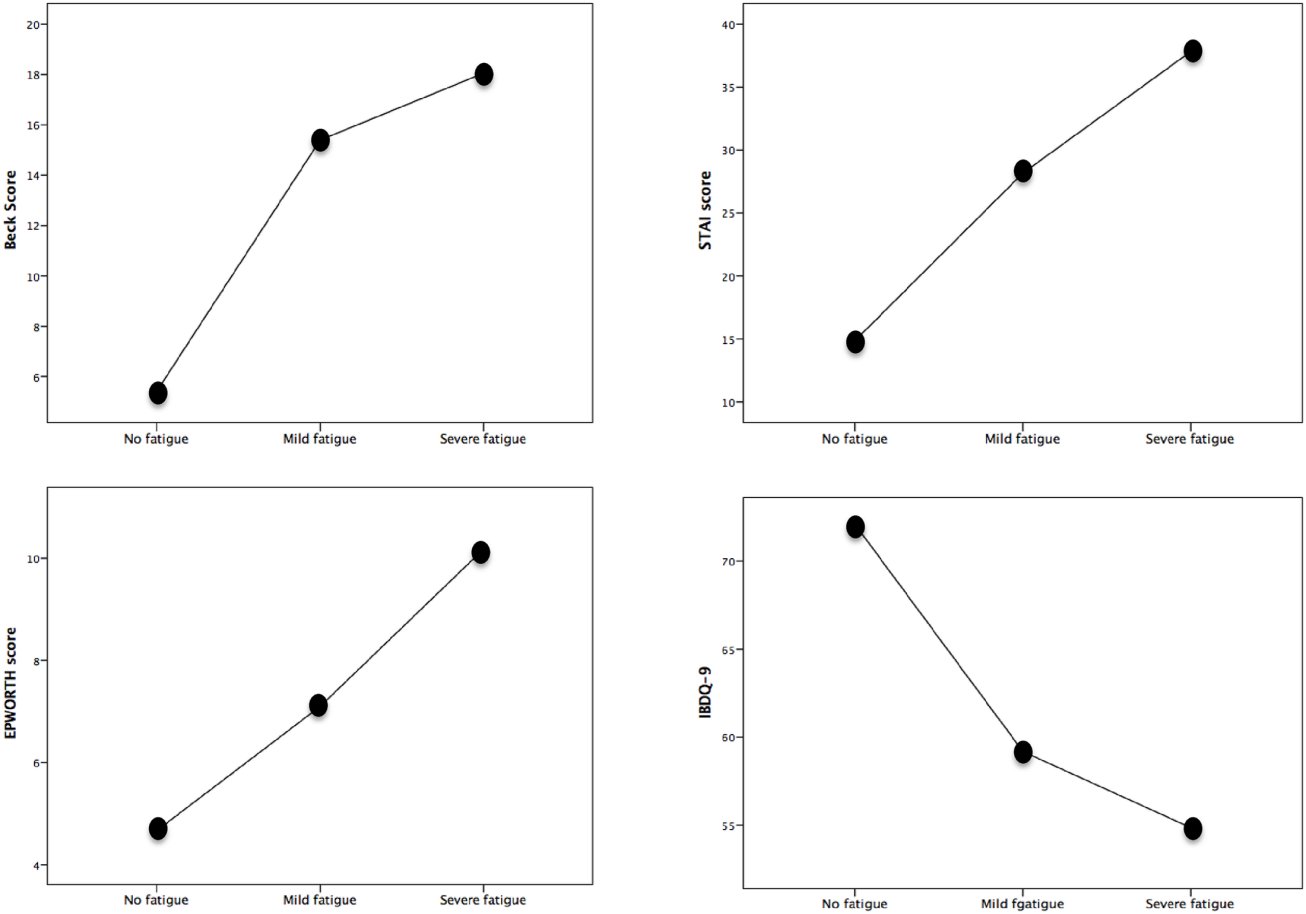
Relationship between psychological test and fatigue.

Patients with fatigue had higher scores on the depression, sleep disturbances and anxiety questionnaires than patients without (Table 3). A strong negative correlation was observed between quality of life (determined by the IBDQ-9 questionnaire) and fatigue (r: 0.81, p< 0.001). Patients with more severe fatigue had worse depression, anxiety, and sleepiness scores (Fig 2) and worse quality of life (p< 0.05 for all).

### 3.4 Multivariate analysis

Finally, multiple regression analysis showed that female sex (OR 4.8; IC 95% 1, 03–22, 70), BMI (OR 1.2; IC 95% 1,01–1,54) and higher depressions core (OR 1.2; IC95% 1,06–1,44) were predictors of increased fatigue. IBDQ-9 scores (OR 0.82; IC 95% 0, 74–0, 93) were inversely and independently related to fatigue.

## 4. Discussion

The present study shows that fatigue is a frequent symptom in patients with IBD even in clinical remission. Major associated factors of fatigue are female sex, high BMI and high scores on depression questionnaires. No relationship was found between the remaining clinical or biological factors determined in our study (interleukins, micronutrients or blood tests) and fatigue. The most important predictive factors for fatigue were psychological factors such as anxiety and depression.

Fatigue is a very common complaint in psychiatric disorders and in chronic diseases like rheumatoid arthritis [2], multiple sclerosis [1] and IBD. Disease activity is one of the most important associated factors in IBD-fatigued patients [3, 4, 19] but the high prevalence of fatigue in asymptomatic patients has demonstrated the need to study other factors. An abstract published by De Haar et al. [11] showed that fatigue in asymptomatic patients was associated with an increase in pro-inflammatory cytokines and with a higher percentage of memory T-cells and neutrophils. In our study, however, assessing the same cytokines (IL-5, 8 and 12) we found no correlation between any of these interleukins and fatigue. Nor did we observe increases in inflammatory factors such as CRP or ESR; therefore we did not find evidence of micro-inflammation as an explanation for fatigue in our IBD patients. Micronutrients were determined to ascertain their relationship with possible deficiency and fatigue in IBD patients. Some previous reports have described multiple micronutrient deficiencies in patients with IBD and have suggested a possible relationship between these deficiencies and the presence of fatigue. Interestingly, we did not find any marked nutrition deficiencies. Micronutrient deficiencies have been reported to be prevalent in patients with IBD even when the disease is in remission [25–27]; our findings probably reflect the fact that disease control in IBD patients has improved markedly in recent years.

Female sex, BMI and joint disorders presented strong associations with fatigue. Even though bowel disease was in remission, many of our patients suffer from inflammatory arthritis or, more frequently, slight joint symptoms. This extra-intestinal activity could play an important role in the presence of fatigue, a common complaint in rheumatologic diseases [28]. In fact, in our series, patients who reported joint symptoms had much lower scores on the FS than those without symptoms (Table 3). Despite this strong relationship, joint disorders were not a predictor of fatigue in the multivariate analysis. This may have been because of the small number of patients affected (21% of all those studied) or because these disorders were strongly associated with other factors such as female sex, age or BMI.

As in previous studies [29, 30], female sex was a strong predictor of fatigue, but no good explanation was found for this association. Crohn’s disease patients were more often fatigued than UC patients. In contrast, when both CD and UC patients were fatigued, they presented similar FS scores.

Psychological factors such as depression was strongly correlated with the presence of fatigue, in the multivariate analysis as well. Anxiety was also related to fatigue but not reaching significance in the multivariate analysis. When we divide groups in mild or severe fatigue, those with more severe symptoms had worse anxiety or depression scores and worse quality of life. Previous studies have reported similar findings. It is very difficult, however, to establish whether anxiety and/or depression are a cause or a consequence of fatigue in IBD patients. Clearly, further research in this area is needed to manage this troublesome symptom.

One limitation of the study is patient selection, since we recruited only patients who were being monitored at the Digestive day care hospital for biological and/or immunosuppressive therapy. Although most of the patients were in full remission, they represent a selected group of patients with more severe disease. It is possible, therefore, that this study overestimated the prevalence of fatigue in the general IBD patient population. Another limitation is that determination of faecal calprotectin was not planned in the original protocol. We selected patients who had been stable for a long time with no clinical activity or minimal unspecific symptoms and normal C-reactive protein (as an objective surrogate of inflammation). Although long term clinical and biological remission may be a rather good predictor for a normal endoscopy the determination of faecal calprotectin would have been useful to further confirm that patients were in complete remission especially in patients with colonic disease.

Nevertheless, our study shows that an important subgroup of patients with IBD had severe fatigue and that the impairment of QoL was serious enough to merit treatment. The identification of associated factors of fatigue may be of help to clinicians when taking therapeutic decisions. Indeed, clinicians should evaluate the possible value of psychological interventions, including pharmacological treatment.

In conclusion, fatigue is a highly prevalent symptom in IBD patients even in clinical remission. None of the organic factors analyzed except female sex and high BMI were associated with fatigue. In contrast, depression presented strong association and appear to be the only factors that can be modified.

## Supporting information

S1 File Data set plos. (SAV)
